# Wastewater Surveillance for Poliovirus in Selected Jurisdictions, United States, 2022–2023 

**DOI:** 10.3201/eid3011.240771

**Published:** 2024-11

**Authors:** Erin R. Whitehouse, Nancy Gerloff, Randall English, Stacie K. Reckling, Mohammed A. Alazawi, Meghan Fuschino, Kirsten St George, Daniel Lang, Eli S. Rosenberg, Enoma Omoregie, Jennifer B. Rosen, Alyse Kitter, Colin Korban, Massimo Pacilli, Trisha Jeon, Joseph Coyle, Russell A. Faust, Irene Xagoraraki, Brijen Miyani, Charles Williams, James Wendt, Sarah M. Owens, Rosemarie Wilton, Rachel Poretsky, Lynn Sosa, Kathy Kudish, Manisha Juthani, Elizabeth F. Zaremski, Susan E. Kehler, Nagla S. Bayoumi, Sarah Kidd

**Affiliations:** Centers for Disease Control and Prevention, Atlanta, Georgia, USA (E.R. Whitehouse, N. Gerloff, R. English, S. Kidd); Agency for Toxic Substances and Disease Registry, Atlanta (S.K. Reckling); New York State Department of Health, Albany, New York, USA (M.A. Alazawi, D. Lang, E.S. Rosenberg); Wadsworth Center, New York State Department of Health, Albany (M. Fuschino, K. St George); New York City Department of Health and Mental Hygiene, Long Island City, New York, USA (E. Omoregie, J.B. Rosen); Chicago Department of Health, Chicago, Illinois, USA (A. Kitter, C. Korban, M. Pacilli); Rush University Medical Center, Chicago (T. Jeon); Michigan Department of Health and Human Services, East Lansing, Michigan, USA (J. Coyle); Oakland County Health Division, Pontiac, Michigan, USA (R.A. Faust); Michigan State University, Lansing, Michigan, USA (I. Xagoraraki, B. Miyani); Illinois Department of Public Health, Springfield, Illinois, USA (C. Williams, J. Wendt); Argonne National Laboratory, Lemont, Illinois, USA (S.M. Owens, R. Wilton); University of Illinois Chicago, Chicago (R. Poretsky); Connecticut Department of Public Health, Hartford, Connecticut, USA (L. Sosa, K. Kudish, M. Juthani); New Jersey Department of Health, Trenton, New Jersey, USA (E.F. Zaremski, S.E. Kehler, N.S. Bayoumi)

**Keywords:** wastewater surveillance, wastewater-based epidemiological monitoring, poliovirus, environmental surveillance, viruses, United States

## Abstract

Wastewater testing can inform public health action as a component of polio outbreak response. During 2022–2023, a total of 7 US jurisdictions (5 states and 2 cities) participated in prospective or retrospective testing of wastewater for poliovirus after a paralytic polio case was identified in New York state. Two distinct vaccine-derived poliovirus type 2 viruses were detected in wastewater from New York state and New York City during 2022, representing 2 separate importation events. Of those viruses, 1 resulted in persistent community transmission in multiple New York counties and 1 paralytic case. No poliovirus was detected in the other participating jurisdictions (Connecticut, New Jersey, Michigan, and Illinois and Chicago, IL). The value of routine wastewater surveillance for poliovirus apart from an outbreak is unclear. However, these results highlight the ongoing risk for poliovirus importations into the United States and the need to identify undervaccinated communities and increase vaccination coverage to prevent paralytic polio.

In June 2022, a case of paralytic polio caused by vaccine-derived poliovirus type 2 (VDPV2) was identified in an unvaccinated adult in Rockland County, New York, USA, historically a county with low vaccination coverage (*1*). In addition, poliovirus type 2 (PV2) genetically linked to the VDPV2 isolated from the patient was detected in wastewater samples from Rockland and several surrounding counties, indicating community transmission. This case was the first known case of paralytic polio in the United States since 2013 (*2*) and the first documented instance of community transmission of poliovirus in the United States since 2005 (*3*).

For decades, the Global Polio Eradication Initiative has used wastewater testing (or environmental surveillance) as a tool to describe the extent of poliovirus circulation when cases of paralytic polio were identified in a community or in at-risk communities with insufficient acute flaccid paralysis surveillance for paralytic polio (*4*,*5*). More recently, countries such as the United Kingdom, Israel, Netherlands, and France have implemented wastewater surveillance for poliovirus in the absence of reported cases of paralytic polio to identify when a community is at risk before a paralytic case occurs (*6*–*9*). Indeed, poliovirus can circulate for an extended period without causing a paralytic case. Among unvaccinated persons, paralysis is estimated to occur in only 1 in 190 to 1 in 1,900 persons infected with poliovirus (depending on poliovirus type) (*10*). Further, inactivated polio vaccine, the only polio vaccine used in the United States since 2000, effectively prevents paralytic disease caused by poliovirus, but it does not prevent gastrointestinal infection or transmission (*11*). It is unknown how common the silent circulation of undetected poliovirus infections is in the United States.

After the paralytic polio case was identified in Rockland County, several surrounding jurisdictions (New York City [NYC], Connecticut, and New Jersey) participated in wastewater testing for poliovirus as part of the outbreak response to describe the geographic extent and duration of poliovirus transmission in the area. Subsequently, Michigan, Illinois, and the city of Chicago, Illinois, piloted poliovirus wastewater-testing projects in their jurisdictions. A previous report summarized results from New York state and NYC through November 2022 (*12*). This report describes the results from all 7 jurisdictions and includes results from samples collected through December 2023.

## Methods

### New York State and NYC

Initial wastewater testing for poliovirus was conducted by New York State Department of Health and NYC Department of Health and Mental Hygiene in collaboration with the Centers for Disease Control and Prevention (CDC) using the existing National Wastewater Surveillance System (NWSS) in NYC and 8 counties in New York state (Orange, Rockland, Nassau, Putnam, Sullivan, Ulster, Suffolk, and Westchester) that were proximal to Rockland County, where the case of paralytic polio was reported. We retrospectively tested stored wastewater samples for poliovirus; samples were originally collected for SARS-CoV-2 surveillance during March 9, 2022–July 25, 2022, in New York state and May 31, 2022–July 20, 2022, in NYC. After the sample from the paralytic case was confirmed positive at CDC on July 21, 2022, we collected and tested wastewater samples from NYC and the 8 New York counties in real time as part of the polio outbreak response through December 2023. Later, additional stored wastewater samples collected during June 2, 2022–December 14, 2022, from 47 other counties in New York were also retrospectively tested to more thoroughly assess the geographic scope of the outbreak.

In New York state, we collected 250 mL of 24-hour time-weighted or flow-weighted samples from the influent of wastewater treatment plants. In NYC, we collected 500 mL of 24-hour flow-weighted composite samples. Samples were collected approximately 1 or 2 times weekly from each site. We processed wastewater samples using either ultracentrifugation or polyethylene glycol precipitation for virus concentration followed by nucleic acid extraction (*13*). During June 2022–March 2023, we forwarded the extracts to the Wadsworth Center (state laboratory for the New York Department of Health) or the New York City Public Health Laboratory, where they were packaged and shipped to CDC. At CDC, we screened total nucleic acids (TNA) for the presence of poliovirus using the pan-poliovirus real-time reverse transcription PCR (rRT-PCR) assay and sequenced positive samples as previously described (*12*,*14*–*16*). We performed genetic linkage of the vaccine-derived poliovirus on the basis of World Health Organization (WHO) and Global Polio Eradication Initiative recommendations (*17*).

To increase sensitivity of the testing after receiving several indeterminate results in 2 sewersheds in NYC, we collected additional 500-mL wastewater specimens at the Newtown Creek–Brooklyn Queens sewershed (August 2022) and in Kings County, Owl’s Head, sewershed (October 2022). Those specimens underwent enterovirus-specific concentration and WHO standard poliovirus isolation methods at CDC; we detected poliovirus presence by rRT-PCR and confirmed serotypes by genomic sequencing (*18*,*19*).

After March 2023, testing for NYC and New York state was performed locally, either at the NYC Public Health Laboratory or the Wadsworth Center. Sampling, processing, and extraction from select sewersheds continued unchanged. We screened extracts on an Applied Biosystems 7500 Fast Dx real-time PCR system (ThermoFisher Scientific, https://www.thermofisher.com) using identical pan-poliovirus rRT-PCR (*16*), after optimization of the primer and probe concentration at each respective laboratory (*15*). We sent extracts from positive samples, defined as a cycle threshold of <37, or indeterminate samples, defined as a cycle threshold of 37–40, to CDC for confirmation and genetic sequencing. Testing in NYC and New York state is ongoing; we present results through December 2023.

### New Jersey and Connecticut

Given their states’ proximity to the paralytic case, New Jersey Department of Health and Connecticut Department of Public Health released stored TNA samples that were collected weekly during May 11, 2022–August 8, 2022, in New Jersey and May 8, 2022–August 3, 2022, in Connecticut for SARS-CoV-2 surveillance as part of NWSS activities to the Polio Response at CDC (*20*). Wastewater samples submitted to NWSS were generally collected as 24-hour flow-weighted or time-weighted composites of ≈150 mL, although occasional post–grit removal or grab samples were collected. CDC tested the TNA for poliovirus using the same methods described previously (*12*,*15*,*16*).

### Pilot Wastewater Surveillance in Additional Jurisdictions

As part of polio emergency response activities, we identified additional jurisdictions to pilot wastewater surveillance for poliovirus in nonoutbreak jurisdictions. Criteria for inclusion were existing participation in the NWSS to provide sufficient structure for reporting results, postal (ZIP) code–level data on vaccine coverage to identify areas with lower vaccine coverage, and identification of appropriately sized sewersheds for testing. Jurisdictions also used data on previous vaccine-preventable disease outbreaks (e.g., measles), a proxy for low vaccination coverage, to prioritize communities for testing (*21*). Illinois Department of Public Health opted for retrospective analysis using archived TNA extracted from wastewater samples in Kankakee, Rock Island, and St. Clair Counties collected during April 2022–April 2023. Chicago Department of Public Health and Michigan Department of Health and Human Services conducted prospective sampling and testing; Chicago officials tested in Cook County, Illinois, during March–July 2023 and Michigan officials conducted testing in Oakland County, Michigan, during June 2023–November 2023.

In Chicago and Illinois, the 24-hour time-weighted or volume-weighted influent composite 100 mL samples of wastewater were processed at the laboratory at the University of Illinois Chicago. After concentration using Ceres Nanotrap A Particles with Enhancement Reagent 1 (Ceres Nanosciences, https://www.ceresnano.com), we extracted RNA using the MagMax Viral Pathogen and Microbiome Ultra kits on the KingFisher Apex (both ThermoFisher Scientific, https://ww.thermofisher.com). We sequenced TNA of prospective Chicago samples at the Rush University Regional Innovative Public Health Laboratory, whereas archived TNA from Illinois samples were sequenced at the Argonne National Laboratory Environmental Sample Preparation and Sequencing Facility. Both laboratories used the methods developed by the Poliovirus Sequencing Consortium (https://polionanopore.org); data were analyzed using the PSC Piranha software package (https://github.com/polio-nanopore/piranha) (*22*,*23*).

Michigan prospectively collected 24-hour time-weighted composite 1-L samples of influent untreated wastewater weekly from 2 locations in Oakland County and processed wastewater samples using polyethylene glycol precipitation followed by nucleic acid extraction. After cDNA synthesis, we tested the samples with conventional PCR targeting the pan-polio viral protein 1 gene (*22*). PCR products from conventional PCR were visualized with gel electrophoresis and Sanger-sequenced. To confirm the sequencing results, all samples were also tested and analyzed with a GT molecular ddPCR Polio Typing Wastewater Surveillance Assay Kit (GT Molecular, https://www.gtmolecular.com) for the Bio-Rad QX200 Droplet Digital PCR System (Bio-Rad Laboratories, https://www.bio-rad.com) following the manufacturer’s protocol. 

Population estimates and percent coverage were reported by the jurisdiction or from CDC NWSS (for New Jersey and Connecticut) based on April 2020 US census data (*24*). This activity was reviewed by CDC, deemed not research, and conducted consistent with applicable federal law and CDC policy (e.g., 45 C.F.R. part 46.102(l)(2), 21 C.F.R. part 56; 42 U.S.C. §241(d); 5 U.S.C. §552a; 44 U.S.C. §3501 et seq).

## Results

### New York State and NYC

**Figure 1:**
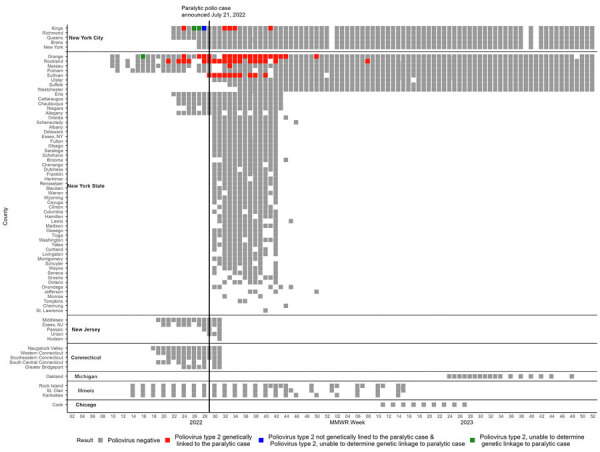
Positive and negative poliovirus results by MMWR week by county and jurisdiction in wastewater surveillance for poliovirus, United States, March 5, 2022–December 31, 2023. Colored squares represent poliovirus results for >1 wastewater samples collected during an MMWR week, including results from prospective testing (New York state, New York City, Chicago, Michigan) and retrospective testing of archived samples (New York state, New York City, Illinois, New Jersey, Connecticut). Any week with a positive poliovirus result is colored red, green, or blue, depending on genetic linkage to the case. Indeterminate results are not included in this figure. For context, the paralytic case was confirmed July 21, 2022 (MMWR week 29), with onset in June 2022. MMWR, Morbidity and Mortality Weekly Report.

In New York state (excluding NYC), 86 samples collected from 7 sewersheds in 4 counties (Nassau, Orange, Rockland, and Sullivan) during May 23, 2022–February 22, 2023, tested positive by pan-poliovirus real-time PCR. Sequencing results confirmed PV2 that was genetically linked to VDPV2 isolated from the paralytic polio patient from Rockland County (Figure 1). In NYC, 6 samples collected during June 14, 2022–October 12, 2022, were PV2-positive and genetically linked to the outbreak. A second, genetically distinct VDPV2 was identified in a sample collected in NYC on July 10, 2022, representing a separate importation event into the United States. New York state also had 1 sample (April 21, 2022; Orange County) and NYC 4 samples (June 28, 2022–July 12, 2022) that tested positive for PV2, but genetic material was insufficient to determine linkage to the outbreak.

**Figure 2:**
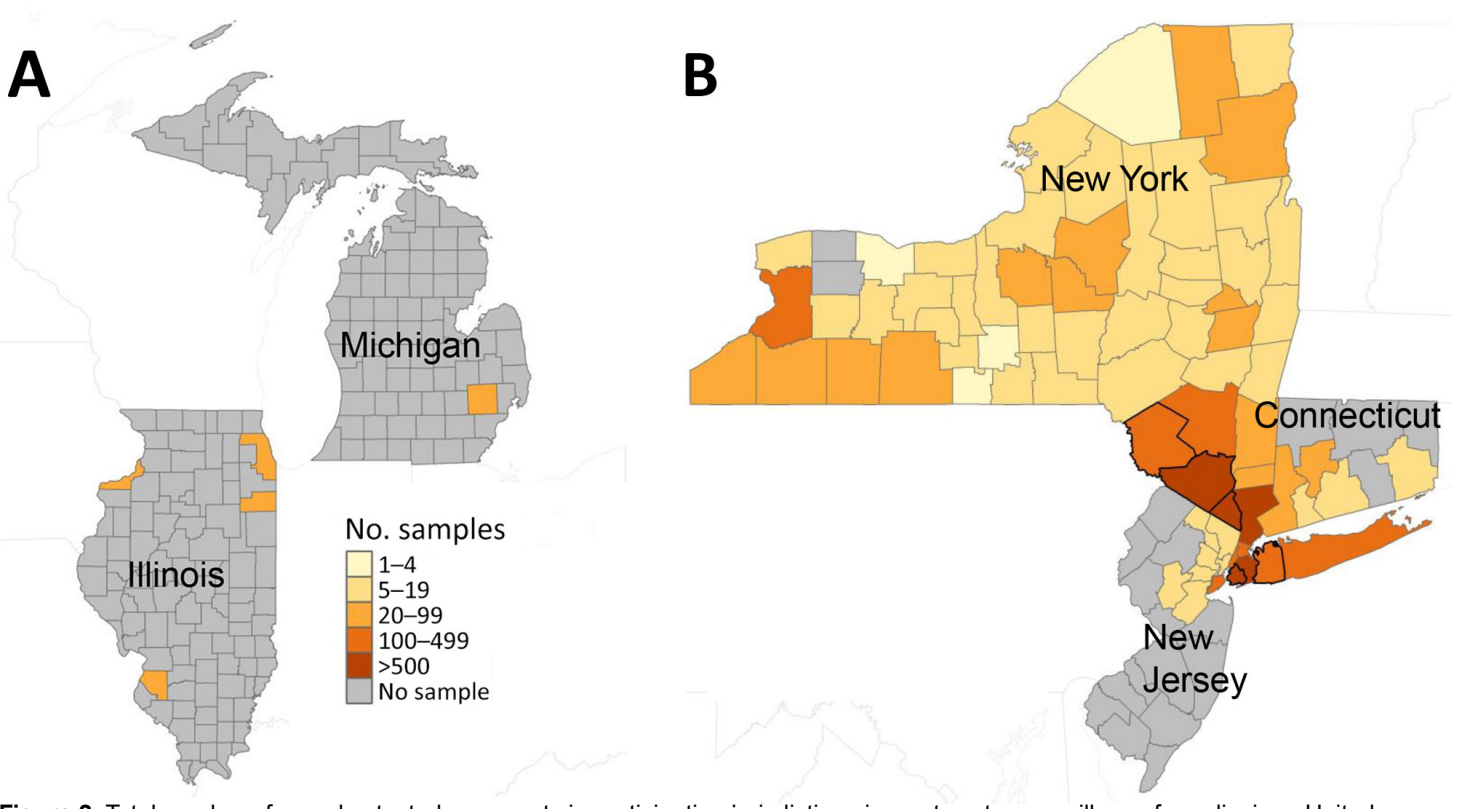
Total number of samples tested per county in participating jurisdictions in wastewater surveillance for poliovirus, United States, March 5, 2022–December 31, 2023. This figure represents the number of samples per county in participating jurisdictions on a logarithmic scale. A) Illinois, including the city of Chicago, and Michigan; B) New York, including New York City; Connecticut; and New Jersey. The thicker black borders in panel B show counties that had a positive sample for poliovirus.

Overall, as a part of the outbreak response in New York state, a total of 3,985 samples across 46 sewersheds from 8 counties were collected during March 9, 2022–December 31, 2023; estimated coverage for each sewershed was 6%–96%, representing ≈2.9 million persons (Figure 2; Appendix Table). In NYC, a total of 1,888 samples across 16 sewersheds were collected during May 31, 2022–December 31, 2023, as a part of the outbreak response; the last positive test was October 2022. Some counties were represented by multiple unique sewersheds, so the coverage was additive (Appendix Table). The estimated combined percentage of population coverage of the sewersheds by county was 96%–99.5% in NYC. This coverage represents an estimated 8.5 million persons.

Subsequently, New York retrospectively tested samples from 47 additional counties that were not included in the original outbreak investigation. In those 47 counties, 1,032 samples collected during June 2, 2022–December 14, 2022, from 77 sewersheds were all negative for poliovirus (Figure 2; Appendix Table). The estimated coverage in this additional retrospective testing for each sewershed was 2%–85%, representing >2.7 million persons across the state.

### New Jersey and Connecticut

We detected no poliovirus in the retrospective samples tested as a part of the outbreak response in New Jersey and Connecticut (Figure 1). In New Jersey, 32 samples collected during May 11, 2022–August 5, 2022, from 5 sewersheds in 7 counties were tested; 3 sewersheds covered multiple counties. The estimated combined percentage of population coverage of the sewersheds by county was 22%–88% and represented ≈3.2 million persons. In Connecticut, 87 samples collected during May 3, 2022–August 3, 2022, from 10 sewersheds in 5 planning regions (i.e., county equivalent in Connecticut) were tested. The estimated percentage of planning region population covered by each sewershed was 22%–47% and represented ≈830,000 persons (Appendix Table).

### Pilot Wastewater Surveillance in Additional Jurisdictions

We detected no poliovirus in wastewater surveillance in the 3 pilot jurisdictions (Figures 1, 2). In Chicago, we tested 36 prospectively collected samples from 4 sewersheds in Cook County during March 5, 2023–July 2, 2023, representing an estimated 4.6 million persons (88% coverage from the sewersheds). In Illinois, we tested 137 samples collected from 7 sewersheds in 3 counties during April 5, 2022–April 5, 2023. The estimated percentage of the county population covered by sewersheds was 48%–66%, a total of ≈300,000 persons. Finally, in Michigan, we tested 44 samples collected from 2 sewersheds in Oakland County during June 12, 2023–November 29, 2023. Those sewersheds represented ≈250,000–320,000 persons (20%–25% county coverage) (Appendix Table).

## Discussion

Wastewater surveillance for poliovirus identified ongoing transmission of poliovirus in Rockland County and adjacent New York counties during an outbreak of poliovirus in the United States (*10*). It also retrospectively demonstrated the presence of poliovirus in the community for several months before the paralytic polio case was identified. Genetic characterization of the detected polioviruses confirmed that almost all wastewater detections were related to the virus isolated from the paralytic polio case. However, wastewater surveillance in NYC also identified a separate importation of VDPV2 that was not genetically related to the New York state case or other outbreaks globally. This second importation was an isolated detection and was not associated with a paralytic case. No related virus has been identified in subsequent testing in NYC or the surrounding counties, indicating that no sustained transmission occurred after that importation. An additional importation of poliovirus was identified in wastewater in Utah by researchers in 2022 and confirmed as poliovirus by CDC (*25*). Subsequent testing from the same site and from the surrounding areas failed to detect additional poliovirus-positive samples. Other researchers have likely tested wastewater for poliovirus in the United States, but CDC was not notified to confirm a positive result (*26*).

No poliovirus was detected in wastewater surveillance by the pilot jurisdictions (Michigan, Illinois, and Chicago) or the retrospective testing of samples from New Jersey, Connecticut, and 47 additional counties in New York. The last poliovirus-positive samples detected in New York state and NYC, where wastewater testing has continued in the outbreak-affected areas, were collected on October 12, 2022, and February 22, 2023. As of October 2024, >2 years after the paralytic case and >1 year after the last positive wastewater detection, no further positive detections have been reported, suggesting the end of the outbreak (*27*,*28*). 

The findings in this report indicate that the United States experiences periodic importations of poliovirus from other countries and is at risk for community transmission, especially in communities that are undervaccinated. However, even though this report was limited to 7 jurisdictions, the data suggest that sustained transmission after importation into the United States is likely a rare event, possibly because of high-quality water and sanitation services and generally high vaccination coverage in most of the country (*29*). Although the transition from oral poliovirus vaccination to inactivated polio vaccine in routine immunization in the United States might enable gastrointestinal poliovirus infection and extensive asymptomatic transmission, no evidence exists of widespread poliovirus transmission in the United States.

The first limitation of this study is that only 80% of US households are on a sewer system that can be sampled by sewershed wastewater surveillance, and this report includes data for just 7 jurisdictions (*30*). Further, outside of New York state, only a few counties in each jurisdiction participated, with limited sewersheds and for a limited period of time. Therefore, these data might not be representative of the United States as a whole, and poliovirus might have been present in wastewater in communities where testing did not occur. In addition, negative results should be interpreted with caution, because sensitivity and limits of detection of PCR testing for poliovirus in wastewater have not yet been defined. Every jurisdiction was responsible for their own test validation and testing methods were not validated by CDC. However, all jurisdictions reported identifying nonpolio enteroviruses. The selection of sewersheds, timing of poliovirus shedding, and the number of infected persons can all affect the ability to detect poliovirus if present. Some sewersheds included in this report had catchment areas that were larger than those recommended by the WHO (≈300,000 persons), potentially affecting the sensitivity of results (*31*,*32*). In addition, varying sewage treatment, concentration, and testing methods might affect the results. Any pretreatment of wastewater might affect the integrity of nucleic acids, giving false-negative results in downstream molecular testing.

As wastewater surveillance for other pathogens continues to expand, the appropriate role of wastewater surveillance for rare diseases like polio in nonendemic areas is still being refined. When used during an outbreak, as in New York, Connecticut, and New Jersey in 2022, wastewater surveillance is a useful tool for identifying the geographic and temporal scope of the outbreak and for identifying the communities at increased risk for poliovirus exposure. Outside of the outbreak setting, several key considerations exist for jurisdictions considering wastewater surveillance for poliovirus. Although instituting a new wastewater surveillance system is challenging, participating jurisdictions found that implementing poliovirus testing within an established wastewater surveillance infrastructure such as NWSS was generally feasible (*30*). However, implementing the additional precautions for poliovirus testing as described by the National Authority for Containment of Poliovirus can be burdensome, not only for health departments but also for laboratories and institutions located within the sewershed areas that are tested (*32*,*33*). Other considerations include ensuring that watershed companies and employees responsible for sample collection are not overburdened with collection processes, that the health and safety of workers are protected, and that any poliovirus detections are communicated clearly to workers and the general public (*32*).

When selecting sewersheds and communities for possible wastewater surveillance for poliovirus, health departments should consider the size of the sewershed catchment area and community perception of being selected for surveillance. A sewershed serving a population that is too small could lead to privacy concerns, whereas sewersheds with larger-than-recommended catchment area populations might compromise test sensitivity. Targeting specific communities for wastewater testing is controversial, and ethical considerations for testing should be taken into account, particularly when specific racial, ethnic, or religious communities might be identified by their sewershed (*32*,*34*,*35*). The same communities that are at risk for polio because of undervaccination might also be at increased risk for stigmatization and health inequities because of ethnic, religious, or racial makeup. Incidental identification of specific counties or communities because of wastewater surveillance could contribute to increased stigmatization (*36*). For those and other reasons, wastewater surveillance might not be acceptable to all communities. Health departments should consider the potential negative consequences of wastewater surveillance and collaborate with at-risk communities when deciding if and where they conduct wastewater surveillance.

Health departments implementing wastewater surveillance for poliovirus should also develop clear communication plans for both negative and positive results to ensure affected populations understand the public health implications and the appropriate action to take, if any. Communication of any poliovirus detections should clarify that poliovirus in wastewater reflects the presence of poliovirus in the community (i.e., not just in sewage material) and should emphasize the importance of vaccination to prevent paralysis. All health departments conducting wastewater surveillance for poliovirus, especially those in communities with poliovirus detections, should anticipate an increase in inquiries about polio vaccination and whether the public needs additional doses. A substantial proportion of those inquiries might come from fully vaccinated persons and persons who are not at increased risk for infection; communication plans should also clarify who is at risk and provide reassurance for those who are not.

Ultimately, the primary purpose of wastewater surveillance for poliovirus is to prevent cases of paralytic polio by identifying communities at increased risk for poliovirus exposure and ensuring high vaccination coverage in those communities. However, how wastewater detections affect public perception of risk, or whether they lead to behavior change (i.e., vaccine uptake), is unclear. In New York state during the 2022 outbreak, publicity surrounding the presence of poliovirus in wastewater did not substantively increase vaccination rates in affected undervaccinated communities (*1*,*12*). Findings from focus groups have highlighted challenges involved in addressing barriers to vaccination (*12*,*34*). Similar challenges with barriers to vaccination and perceived low risk for poliovirus infection among communities with lower vaccine coverage were noted in the United Kingdom during a 2022 response to multiple detections of VDPV2 in sewage (*37*). Certainly, identifying undervaccinated communities and improving routine vaccination coverage are public health priorities. This work can and should be ongoing before, or even without, a wastewater detection.

Wastewater surveillance has the potential to be a vital public health tool for monitoring disease and promoting public health action in certain situations, as seen in the COVID-19 pandemic and during the outbreak response to the 2022 paralytic polio case in New York. However, outside of the outbreak setting, considering the public health resources required for ongoing surveillance and whether the data will lead to public health action are key (*38*). As long as poliovirus is circulating elsewhere in the world, periodic importations into the United States are to be expected (*7*). Regardless of wastewater surveillance availability in jurisdictions, vaccination, the most effective public health intervention to prevent paralytic polio, is readily available in the United States. State and local health departments should identify communities with low vaccination coverage and collaborate with those communities to improve vaccination rates to prevent paralytic polio and other vaccine preventable diseases.

Appendix Additional information about wastewater surveillance for poliovirus in selected jurisdictions, United States, 2022–2023.
