# Supplementary material for: Wastewater Surveillance for Poliovirus in Selected Jurisdictions, United States, 2022–2023
Source: Emerg Infect Dis. 2024 Nov;30(11):2279–87. doi: 10.3201/eid3011.240771 (PMC11521156; doi:10.3201/eid3011.240771)
Supplement: Appendix — Additional information about wastewater surveillance for poliovirus in selected jurisdictions, United States, 2022–2023. [file 24-0771-Techapp-s1.pdf]

Article DOI: https://doi.org/10.3201/eid3011.240771

EID cannot ensure accessibility for supplementary materials supplied by authors. Readers who have difficulty accessing supplementary content should contact the authors for assistance.

Wastewater Surveillance for Poliovirus in Selected Jurisdictions, United States, 2022–2023

Appendix

Appendix Table. The total number of counties, sewersheds, estimated coverage of the sewersheds and the estimated number of people represented, dates of sample collection, and total number of samples in participating jurisdictions, March 5, 2022–December 31, 2023*

Jurisdiction	County/Planning Region(s)†	No. sampling sites	County Population (April 2020)	Estimated percentage of county population covered by sewershed(s)	Estimated number of people represented by the sewershed(s)	Date range of sample collection	Total no. samples tested
Chicago	Cook	4	5275555	88.0%	4642488	3/12/2023–7/2/2023	36
Connecticut	Connecticut Metropolitan/Greater Bridgeport	2	325774	46.7%	152265	6/13/2022–8/3/2022	10
Connecticut	Naugatuck Valley	2	450435	29.2%	131500	5/3/2022–8/3/2022	24
Connecticut	South Central Regional	1	570455	40.0%	228000	5/10/2022–8/2/2022	9
Connecticut	Southeastern Connecticut	2	280428	22.1%	62000	5/24/2022–8/3/2022	15
Connecticut	Western Connecticut	3	620519	41.9%	260000	5/8/2022–8/3/2022	29
Illinois	Kankakee	1	107510	52.4%	56324	4/5/2022–4/3/2023	22
Illinois	Rock Island	2	144667	47.7%	68963	7/12/2022–4/5/2023	42
Illinois	St. Clair	4	257412	66.1%	170201	4/5/2022–4/4/2023	73
Michigan	Oakland	2	1274402	20%–25%	254880–318600	6/12/2023–11/29/2023	44
New Jersey	Bergen, Essex, Hudson, Passaic	1	3068423	21.7%, 59.8%, 68.3%, 76.4%	1620409	5/16/2022–8/5/2022	10

Jurisdiction	County/Planning Region(s)†	No. sampling sites	County Population (April 2020)	Estimated percentage of county population covered by sewershed(s)	Estimated number of people represented by the sewershed(s)	Date range of sample collection	Total no. samples tested
New Jersey	Essex, Union	1	1438131	28.3%, 43.9%	496638	7/18/2022–8/3/2022	4
New Jersey	Hudson	1	724858	14.8%	107113	8/2/2022	1
New Jersey	Middlesex, Somerset, Union	1	1783918	86.3%, 33.3%, 12.5%	932171	5/11/2022–8/3/2022	14
New Jersey	Passaic	1	525054	10.4%	54642	7/12/2022–8/2/2022	3
New York City	Bronx	1	1472653	52.2%	769120	7/5/2022–12/26/2023	123
New York City	Bronx, New York	1	3166903	46.2%, 28.9%	1197129	7/5/2022–12/26/2023	124
New York City	Kings	4	2736119	76.1%	2082806	5/31/2022–12/26/2023	520
New York City	Kings, New York, Queens	3	6835794	22.4%, 31.9%, 5.9%	1197476	5/31/2022–12/26/2023	229
New York City	New York	1	1694250	38.7%	655795	7/5/2022–12/26/2023	124
New York City	Queens	4	2405425	91.4%	2199116	5/31/2022–12/26/2023	504
New York City	Richmond	2	495752	96.2%	477051	5/31/2022–12/26/2023	264
New York State	Albany	2	314851	31.6%	99493	07/27/2022 - 10/17/2022	26
New York State	Allegany	2	46450	11.4%	5295	06/07/2022 - 10/19/2022	22
New York State	Broome	2	198683	58.3%	115832	08/10/2022 - 11/01/2022	13
New York State	Cattaraugus	1	77034	23.1%	17795	06/06/2022 - 10/25/2022	22
New York State	Cayuga	1	76253	41.7%	31798	07/26/2022 - 10/18/2022	11
New York State	Chautauqua	2	127657	35.3%	45063	06/07/2022 - 10/25/2022	39
New York State	Chemung	1	84142	45.2%	38032	11/02/2022 - 11/02/2022	1
New York State	Chenango	1	47220	14.3%	6752	07/27/2022 - 10/19/2022	12
New York State	Clinton	2	79838	27.5%	21955	08/02/2022 - 10/14/2022	18
New York State	Columbia	1	61560	10.4%	6402	07/26/2022 - 10/18/2022	11
New York State	Cortland	1	46800	57.6%	26957	07/26/2022 - 10/18/2022	10
New York State	Delaware	1	44311	8.1%	3589	07/27/2022 - 10/19/2022	13

Jurisdiction	County/Planning Region(s)†	No. sampling sites	County Population (April 2020)	Estimated percentage of county population covered by sewershed(s)	Estimated number of people represented by the sewershed(s)	Date range of sample collection	Total no. samples tested
New York State	Dutchess	1	297021	12.9%	38316	07/27/2022 - 10/19/2022	21
New York State	Erie	6	954231	82.4%	786286	06/02/2022 - 10/27/2022	283
New York State	Essex	2	37374	25.0%	9344	07/25/2022 - 10/19/2022	27
New York State	Franklin	2	47573	25.4%	12084	07/26/2022 - 10/19/2022	28
New York State	Fulton	1	53333	43.8%	23360	07/25/2022 - 10/17/2022	13
New York State	Greene	1	47932	10.2%	4889	08/18/2022 - 10/20/2022	7
New York State	Hamilton	1	5105	2.2%	112	08/03/2022 - 10/21/2022	11
New York State	Herkimer	2	60144	35.7%	21471	07/25/2022 - 10/17/2022	12
New York State	Jefferson	3	116711	46.2%	53920	09/13/2022 - 12/14/2022	7
New York State	Lewis	1	26587	14.9%	3961	08/03/2022 - 11/09/2022	11
New York State	Livingston	2	61840	33.0%	20407	08/09/2022 - 10/18/2022	19
New York State	Madison	5	68017	34.6%	23534	08/11/2022 - 10/20/2022	39
New York State	Monroe	2	759430	26.1%	198211	08/08/2022 - 09/27/2022	2
New York State	Montgomery	1	49527	39.7%	19662	07/27/2022 - 09/28/2022	10
New York State	Nassau	5	1395767	85.0%	1186402	03/09/2022 - 12/27/2023	496
New York State	Niagara	1	212666	22.7%	48275	06/20/2022 - 10/29/2022	18
New York State	Oneida	2	232113	50.2%	116521	07/26/2022 - 11/03/2022	38
New York State	Onondaga	6	476523	85.0%	405045	07/24/2022 - 11/06/2022	25
New York State	Ontario	1	112485	15.9%	17885	08/09/2022 - 10/18/2022	7
New York State	Orange	9	401315	46.0%	184605	03/09/2022 - 12/29/2023	1120
New York State	Oswego	1	117528	12.1%	14221	07/27/2022 - 10/19/2022	11
New York State	Otsego	1	58528	33.1%	19373	07/27/2022 - 10/20/2022	13
New York State	Putnam	2	97678	6.4%	6251	03/16/2022 - 12/27/2023	98

Jurisdiction	County/Planning Region(s)†	No. sampling sites	County Population (April 2020)	Estimated percentage of county population covered by sewershed(s)	Estimated number of people represented by the sewershed(s)	Date range of sample collection	Total no. samples tested
New York State	Rensselaer	1	161124	49.5%	79756	07/27/2022 - 10/19/2022	12
New York State	Rockland	6	338337	96.1%	325142	03/09/2022 - 12/28/2023	597
New York State	Saratoga	1	235502	64.7%	152370	07/27/2022 - 10/20/2022	13
New York State	Schenectady	1	158052	49.6%	78394	07/26/2022 - 11/17/2022	25
New York State	Schoharie	1	29718	17.9%	5320	07/27/2022 - 10/19/2022	14
New York State	Schuyler	1	17894	20.4%	3650	08/04/2022 - 10/20/2022	9
New York State	Seneca	1	33816	20.6%	6966	07/27/2022 - 10/20/2022	8
New York State	St. Lawrence	1	107733	9.9%	10666	10/05/2022 - 10/05/2022	1
New York State	Steuben	4	93571	24.7%	23112	07/26/2022 - 10/20/2022	82
New York State	Suffolk	8	1525894	21.9%	334171	08/15/2022 - 12/27/2023	285
New York State	Sullivan	6	78613	23.1%	18160	07/21/2022 - 12/28/2023	228
New York State	Tioga	1	48452	7.7%	3731	07/27/2022 - 10/19/2022	11
New York State	Tompkins	1	105737	53.9%	56992	08/31/2022 - 09/07/2022	2
New York State	Ulster	3	181841	22.5%	40914	07/27/2022 - 12/27/2023	219
New York State	Warren	1	65737	35.7%	23468	07/25/2022 - 10/17/2022	12
New York State	Washington	1	61297	20.0%	12259	07/27/2022 - 10/20/2022	11
New York State	Wayne	1	91286	9.8%	8946	07/26/2022 - 10/18/2022	9
New York State	Westchester	7	1004469	83.9%	842749	08/28/2022 - 12/31/2023	942
New York State	Wyoming	1	40544	8.5%	3446	07/26/2022 - 10/18/2022	12
New York State	Yates	1	24769	21.6%	5350	07/27/2022 - 10/19/2022	11

*Note: Population estimates and percent coverage were reported by the jurisdiction or from CDC NWSS (NJ, CT) based on April 2020 U.S. census data.
†Some sewersheds covered multiple counties/planning region(s) so there might be multiple counties/planning region(s) represented and the estimated percentage of the county population covered by the sewershed(s) is separated by county/planning region.
